# Laparoscopic Nephroureterectomy and Management of the Distal Ureter: A Review of Current Techniques and Outcomes

**DOI:** 10.1155/2009/721371

**Published:** 2009-01-08

**Authors:** Davis P. Viprakasit, Amanda M. Macejko, Robert B. Nadler

**Affiliations:** Department of Urology, Feinberg School of Medicine, Northwestern University, Chicago, IL 60208, USA

## Abstract

Laparoscopic nephroureterectomy (LNU) is becoming an increasingly common alternative treatment for transitional cell carcinoma (TCC) of the renal pelvis and ureter due to decreased perioperative morbidity, shorter hospitalization, and comparable oncologic control with open nephroureterectomy (ONU). Mobilization of the kidney and proximal ureter may be performed through a transperitoneal, retroperitoneal, or hand-assisted approach. Each technique is associated with its own benefits and limitations, and the optimal approach is often dictated by surgeon preference. Our analysis of the literature reflects equivalent cancer control between LPN and OPN at intermediate follow-up with significantly improved perioperative morbidity following LPN. Several methods for bladder cuff excision have been advocated, however, no individual technique for management of the distal ureter proved superior. Overall, complete en-bloc resection with minimal disruption of the urinary tract should be optimized to maintain oncologic outcomes. Longer follow-up and prospective studies are needed to fully evaluate these techniques.

## 1. Introduction

Transitional cell carcinoma (TCC) of the renal pelvis and ureter is a disease associated with high propensity for tumor recurrence and progression. Open radical nephroureterectomy (ONU) with bladder cuff excision is the traditional standard treatment for most localized diseases of the upper urinary tract because of its aggressive nature, as well as the difficulty encountered with surveillance of the upper tract urothelium [1]. To obtain adequate exposure, open excision of the distal ureter and bladder cuff requires either two skin incisions or an extended flank incision. This is associated with increased perioperative morbidity and recovery time. First described by Clayman et al. in 1991 [2], laparoscopic radical nephroureterectomy (LNU) has shown significant advantages in terms of blood loss, postoperative pain and recovery time, as well as comparable short and intermediate-term oncologic outcomes with the open treatment [3]. As more urologists are gaining increased comfort with minimally invasive techniques, LNU exhibits a large growth in worldwide popularity [4]. However, the optimal laparoscopic approach for nephroureterectomy as well as the technique for addressing the bladder cuff is unclear. We present a review of the most recent literature detailing the perioperative and cancer control outcomes observed with the various methods of LNU and discuss the reported variations on bladder cuff excision.

## 2. Laparoscopic Nephroureterectomy

### 2.1. Approach

A variety of techniques have been utilized to perform mobilization of the kidney and proximal ureter. These include conventional transperitoneal, conventional retroperitoneal, hand-assisted transperitoneal, and hand-assisted retroperitoneal approaches. The choice of laparoscopic approach is most dependent on the comfort level and training of the surgeon. However, each technique has its own potential advantages. Transperitoneal exposure offers the largest working area and may be beneficial for extensive tumors or lymphadenopathy. The retroperitoneal approach, however, involves decreased bowel manipulation and potentially allows for earlier recovery of bowel function. Additionally, this method may be favored in morbidly obese patients with an obstructing pannus or in patients with a history of previous transperitoneal surgeries [5]. Hand-assistance techniques allow for continued tactile sensation and may lessen the learning curve required in laparoscopy [6]. Most of the experiences with LNU are primarily reported as single-institutional retrospective series. To date, there have been no prospective randomized studies that compare the different techniques for LNU. However, data published in the laparoscopic radical nephrectomy literature may be applicable. For example, a randomized, prospective study comparing conventional transperitoneal, retroperitoneal, and hand-assisted transperitoneal laparoscopic radical nephrectomy was recently published [7]. The hand-assisted technique resulted in a significantly shorter operative time but an increased risk of hernia formation; conversely, conventional transperitoneal surgery was associated with significantly improved perioperative morbidity. Desai et al. reported shorter operative times and faster control of the renal vasculature with retroperitoneal approaches in a prospective randomized comparison of conventional transperitoneal and retroperitoneal laparoscopic radical nephrectomy but no significant differences in other perioperative measures [5].

### 2.2. Perioperative Outcomes

Rassweiler et al. performed a literature review of published studies between 1991 and 2004 of LNU and ONU, including nine comparative studies and 1365 overall patients [3]. As compared to ONU, LNU was associated with a slightly longer operative time (277 versus 220 minutes), significantly lower blood loss (241 versus 463 mL), and shorter hospital stays, but showed similar complication rates (18% versus 21%).

Table 1 summarizes the perioperative outcomes of 12 LNU studies published since 2005. The mean operating time ranged from 165 to 395 minutes (mean 271) in the laparoscopic group (*n* = 465 patients), and 155 to 313 minutes (mean 237) in the open group (*n* = 268 patients). Significantly increased operative time with laparoscopy was noted in three of eight comparative studies, all of which involved the retroperitoneal approach. The blood loss averaged between 183 to 497 mL (mean 279) in the laparoscopic group as compared to a range of 296 to 558 mL (mean 402) in the open group. Significantly less blood loss after laparoscopy was noted in four of eight comparative studies with a similar trend in three of the remaining studies. The overall complication rate ranged from 0 to 37% (mean 18%) in the laparoscopy group and from 0 to 15% (mean 7%) in the open group. The open conversion rate associated with laparoscopy ranged from 0 to 10%. The absolute duration of hospital stays varied between institutions. Out of eight comparative studies, hospitalization following laparoscopy was shorter in seven series with a significant difference in five.

### 2.3. Oncologic Outcomes

The highly aggressive natural history of upper tract TCC, particularly with high-grade and high-stage disease, likely contributes to its increased potential for recurrence and poor prognosis irrespective of the surgical technique. However, there are many concerns that technical aspects of LNU, particularly with regard to management of the distal ureter and bladder cuff, may affect recurrence risks in the bladder, locally, or as port-site metastases secondary to tumor seeding. Rassweiler et al. noted no significant difference in bladder recurrence (24% versus 25%), local recurrence (4% versus 6%) and distant metastases (15.5% versus 15.2%) in eight LNU series and 11 ONU series [3]. The 2-year cancer specific survival rates were also similar (75% versus 76%).

Table 2 summarizes the short and intermediate oncologic outcomes of 14 LNU studies published since 2005. The most common method for bladder cuff excision was an open approach. At a minimum median follow-up of at least 2 years, bladder recurrence after LNU (*n* = 488 patients) ranged from 10 to 55% (mean 30%) as compared to 15 to 55% (mean 33%) in the ONU series (*n* = 512 patients). Local recurrence in the retroperitoneum was documented as 0 to 13% (mean 3.3%) in the laparoscopic (*n* = 588 patients) and 0 to 8% (mean 2.5%) in the open series (*n* = 512 patients). Distant metastases occurred in 0 to 18% (mean 9.5%) of patients undergoing LNU (*n* = 588 patients) as compared to 5 to 35% (mean 14.5%) in the open series (*n* = 512 patients). In six series reporting 2-year disease-specific survival, the rates ranged from 64 to 91% (mean 83%) in the laparoscopic series (*n* = 274 patients) and between 58 to 93% (mean 83.6%) after ONU (*n* = 242 patients). In the five series reporting 5-year survival rates, the outcomes ranged from 68 to 90% (mean 85%) after LNU (*n* = 202 patients) and 62 to 86% (mean 75%) in the ONU groups (*n* = 191 patients). There was no significant difference between LNU and ONU survival rates in the 10 comparative studies. However, absolute comparisons between the surgical approaches are difficult as the percentage of patients with high-grade disease and the follow-up period varied considerably, reflecting a large limitation with the retrospective nature of these studies. In addition, the inclusion of patients with a prior or concomitant history of bladder cancer may affect the oncologic outcome of treating upper tract TCC. In the 14 LNU studies, only 7 addressed this parameter in defining their patient characteristics. The 4 comparative studies including such patients and the 1 study which excluded patients with bladder TCC did not show a significant difference between ONU and LNU groups. However, it is unclear if the underlying biology and tumor aggressiveness in patients with both upper and lower tract TCC differ from patients with isolated upper tract TCC; inclusion of such patients in studies with already relatively low numbers further complicates comparisons of treatment approach.

### 2.4. Port-Site Metastases

One unique concern reported following laparoscopic surgery is the occurrence of recurrent malignant disease at the port-site [22]. To the best of our knowledge, there have been 18 cases published in the literature of port-site metastases of upper tract TCC after laparoscopy (Table 3). In seven cases, the diagnosis of TCC was not suspected preoperatively which influenced the surgical technique. Metastases occurred 3 to 15 months postoperatively (mean 6.8). These overall experiences emphasize that general preventive measures should be undertaken at the conclusion of the surgery including the use of an impermeable organ bag, minimal tissue handling, and the avoidance of gross violation of the urinary system until the specimen has been removed en bloc.

## 3. Bladder Cuff Excision

There is no consensus as to the optimal technique to excise the distal ureter and ipsilateral bladder cuff [1]. Definitive steps to minimize tumor seeding and complete excision of the ureter are mandatory given the 30% to 64% tumor recurrence rate reported following inadequate distal resections [31, 32]. As noted in Table 2, one of the most utilized approaches involves an open approach. This may be accomplished transvesically or extravesically via a lower midline, Pfannenstiel, or Gibson incision, or by incorporation of the hand port incision following hand-assisted LNU. This technique is similarly employed during ONU and offers the surgeon familiarity, direct visualization, and a simultaneous site for en bloc specimen extraction. Awareness of the contralateral trigone and ureteral orifice location should be undertaken as potential injury may occur during ipsilateral dissection or bladder cuff closure [33].

Alternatively, numerous endoscopic approaches have been promoted. In 1952, McDonald et al. described the first endoscopic method of bladder cuff excision, the “pluck” technique, via transurethral resection of the ureteral orifice (TURUO) at the onset of surgery [34]. Resection of the orifice and intramural ureter, however, may require patient repositioning and when performed at the onset of the procedure can expose the extravesical space to potential tumor seeding. Several modifications have been described to this technique in contemporary series, including delaying resection until after kidney mobilization, performing transvesical endoscopy [35] or using transurethral cystoscopy with a Bugbee electrode [36] or Collins knife for excision [37]. Ko et al. reviewed their experience comparing open dissection (*n* = 27 patients) with modified TURUO using a Collins knife (*n* = 19 patients) following nephroureterectomy [38]. At a mean follow-up of over 22 months, they noted similar bladder recurrence rates (22.2% versus 26.3%) without evidence of pelvic recurrence.

The technique of ureteral intussusception has also been described and involves endoscopic extraction of the ligated ureter using a “stripping” method with the assistance of a ureteral catheter [39]. However, this approach is contraindicated with concomitant bladder or ureteral tumors and was noted to have an incomplete excision rate of 18.7% in a large single institutional series of 32 patients [40].

Gill et al. described the method of cystoscopic detachment and ligation which incorporates intramural ureteral dissection with a Collins knife aided by two transvesical laparoscopic ports and an endoloop to ligate the ureteral lumen and minimize potential tumor spillage [41]. While this method most closely echoes the intentions of ONU, it is associated with a steep learning curve and long procedural time [42].

Laparoscopic stapling of the distal ureter and bladder cuff with either cystoscopic unroofing or a pure extravesical approach has also been utilized [43]. This technique, however, has been associated with the potential risk of stone formation [44] or viable tumor cells within the incorporated staple line [45]. A comparison of laparoscopic stapling (20% of cohort) with cystoscopic detachment and ligation (60% of cohort) by Matin and Gill was notable for a positive surgical margin rate of 25% versus 2.8% [46]. Hattori et al. reported their experience between laparoscopic stapling and open bladder cuff excision [44]. They noted a significantly decreased operative time with laparoscopic stapling with no significant difference in bladder and extravesical recurrence-free rates and disease-specific survival at 3-year follow-up. Tsivian et al. detailed a modified technique for excising the periurethral bladder cuff en bloc using a LigaSure Atlas device instead of a stapler [47]. Similarly, excision via harmonic scalpel has also been utilized [48]. Division of the bladder cuff using hemostatic diathermy devices may address the potential concerns of viable tumor cells and stone formation associated with laparoscopic stapling although further study is needed.

While there have been no randomized prospective trials comparing the management of the distal ureter, several groups have reported their retrospective results with several different approaches. In a large multicenter American and European study, Abou El Fettouh et al. noted that the local recurrence rates and the development of metastases depended on pathologic tumor stage and was irrespective of bladder cuff approach (open, TURUO, cystoscopic detachment and ligation, laparoscopic stapling) [49]. In a series of 55 patients undergoing hand-assisted transperitoneal LNU, Brown et al. noted increased perioperative morbidity and complications with TURUO. However, higher positive surgical margins were observed following laparoscopic stapling (29%) or extravesical harmonic scalpel excision (10%) as compared to TURUO or open techniques [50]. Additionally, in patients without active or recent lower tract TCC, concerns exist regarding the increased potential risk of local recurrence when the cystotomy is not primarily closed following excision of the ureteral orifice and bladder neck as with many of the endoscopic approaches. Brown et al. noted their sole pelvic recurrence occurred in 1 of 7 patients without cystotomy closure, leading the authors to also advocate routine bladder defect closure [50]. However, Kurzer et al. reported no local recurrences in 49 patients treated with a modified TURUO technique and no cystotomy closure at a median follow-up of 10 months [51].

## 4. Role of Lymphadenectomy

Given its aggressive nature, the presence of nodal involvement in TCC of the renal pelvis and ureter is a poor prognostic factor and has shown limited response to adjuvant therapies [52]. However, the role and utilization of routine lymph node dissection (LND) in conjunction with either LNU or ONU is not well established. This contrasts lower urinary tract TCC in which extended pelvic LND is well supported in the literature for improved staging and survival benefits [53]. One reason for the variable use of LND (Table 2) is that the standard template for regional lymph node involvement in upper tract TCC has not been well delineated. In a recent review of 42 of 181 patients with upper tract TCC metastases, Kondo et al. noted that the location of lymph node metastases depended on the laterality and level of the primary tumor [54]. Based on their findings, the authors advocated a relatively wide LND template, particularly on the right side to include the paracaval, retrocaval, and interaortocaval nodes. In a follow-up study, the authors noted an improved cancer-specific survival in patients with advanced disease (stage pT3 or higher) undergoing LND although no difference was noted when all pathologic stages were considered [55]. Brausi et al. similarly reported an improved disease-specific survival benefit in patients treated with ONU and LND (81.6%) as compared to ONU alone (44.8%) [56]. However, the retrospective study may have been influenced by a potential bias in patient selection for LND [57]. Additionally, regardless of the benefits of LND, concerns remain regarding the technical challenge of laparoscopic lymphadenectomy. Hattori et al. reported a significantly decreased number of lymph nodes removed following LNU (8.2–11.6) as compared to ONU (16.5) [44]. Busby and Matin, however, reported their experience that removal of an equivalent number of nodes could be performed with both laparoscopic and open approaches [58].

## 5. Future Developments

With the increased popularity in robotic-assisted laparoscopy in urology, individual case reports and small case series have recently described robotic-assisted LNU with either retroperitoneal or transperitoneal approaches [59–62]. The improved dexterity, precision, and control of robotic assistance may better facilitate handling of the distal ureter and bladder cuff [60]. However, concerns with cost and the potential need for patient repositioning and robot redocking may influence the widespread utilization of robotics in treating upper tract TCC.

## 6. Conclusions

Following the increased popularity of laparoscopy in urologic surgery, LNU has become a common treatment for TCC of the renal pelvis and ureter with decreased perioperative morbidity, shorter hospitalization, as well as comparable oncologic outcomes and survival rates as with ONU. The optimal technique for mobilization of the kidney and proximal ureter, as well as excision of the distal ureter and bladder cuff, is still evolving and largely based on surgeon preference. The classic open approach for distal ureter removal is most comparable to the established principles of open oncologic surgery and simultaneously allows for intact en bloc specimen removal. Regardless of technique used, minimal disruption of the urinary tract should be maintained to decrease the risk of recurrences and port-site metastases. The role of routine lymphadenectomy and the utilization of robotic assistance in upper urinary tract TCC are still to be determined. Long-term studies with prospective, randomized trials are necessary to fully evaluate the outcomes of LNU in the management of this aggressive disease.

## Figures and Tables

**Table 1:** Perioperative characteristics of LPN versus ONU cases. CR, conventional retroperitoneal; CT, conventional transperitoneal; HAT, hand-assisted transperitoneal; NL, not listed; ONU, open nephroureterectomy.

Author	Surgery	Number	High grade (%)	OR duration (min)	Blood loss (min)	Conversion (%)	Complication (%)	Hospital days
Muntener et al. [8]	CT	39	31 (80)	312	300	4 (10)	12 (31)	4

Schatteman et al. [9]	CT	100	48 (48)	192	234	7 (7)	19 (19)	10

Rouprêt et al. [10]	CT	20	8 (40)	165	275	1 (5)	3 (15)	4
	ONU	26	19 (73)	155	338	—	4 (15)	9

Okegawa et al. [11]	CR	25	6 (24)	299	258	1 (4)	4 (16)	11
	ONU	23	7 (30)	313	403	—	3 (13)	13

Tsujihata et al. [12]	CR	25	5 (20)	306	322	0 (0)	0 (0)	2
	ONU	24	12 (50)	271	558	—	0 (0)	4

Taweemonkongsap et al. [13]	CR	31	13 (42)	259	289	0 (0)	2 (6)	9.3
	ONU	29	19 (66)	191	314	—	2 (7)	8.7

Chung et al. [14]	HAT	39	16 (41)	233	183	0 (0)	5 (13)	7
	ONU	36	15 (42)	220	422	—	3 (8)	9

Raman et al. [15]	HAT	38	15 (40)	244	191	0 (0)	4 (11)	5
	ONU	52	19 (37)	243	478	—	2 (4)	7

Wolf et al. [16]	HAT	53	26 (49)	279	330	1 (2)	20 (37)	4

Cannon et al. [17]	HAT	34	NL	317	252	0 (0)	9 (26)	8

Chung et al. [18]	HAR	25	11 (44)	252	212	0 (0)	3 (12)	6.5
	ONU	41	17 (41)	212	408	—	3 (7)	9

Nakashima et al. [19]	HAR	36	18 (50)	395	497	1 (3)	11 (31)	18.8
	ONU	37	13 (35)	289	296	—	2 (5)	19.1

**Table 2:** Oncologic outcomes of LPN versus ONU cases. CR, conventional retroperitoneal; CT, conventional transperitoneal; HAR, hand-assisted retroperitoneal; HAT, hand-assisted transperitoneal; LND, lymph node dissection; LS, laparoscopic stapling; NL, not listed; ONU, open nephroureterectomy; SD, surgeon discretion; TURUO, transurethral resection; TV, transvesical.

Author	Surgery	N	Follow up (mo)	History of bladder tumor specified	Bladder cuff surgery	Path stage (Ta,Tis,T1/T2, T3,T4)	Path grade (1/2/3)	LND	Urothelial recurrence (%)	Local recurrence (%)	Port-site recurrence (%)	Distal recurrence (%)	Disease-specific survival (@ year)
Muntener et al. [8]	CT	39	74	Yes	Open (19), LS (13), other (5)	22/15/2NL	8LG/31HG	No	16 (41)	2 (5)	0 (0)	7 (18)	68 (5)

Schatteman et al. [9]	CT/CR	95/5	20	No	Open (55), Other (45)	54/46	24/28/48	20% of cases	NL	13 (13)	3 (3)	16 (16)	88 (2)

Rouprêt et al. [10]	CT	20	69	No	Open	15/511/15	12LG/8HG	SD	2 (10)	0 (0)	0 (0)	2 (10)	90 (5)
	ONU	26	78				7LG/19HG		4 (15)	0 (0)		9 (35)	62 (5)

Okegawa et al. [11]	CR	25	24	Yes	Open	14/119/14	2/17/6	No	5 (20)	0 (0)	0 (0)	2 (8)	91 (2)
	ONU	23	29				1/15/7		4 (17)	0 (0)		3 (13)	89 (2)

Tsujihata et al. [12]	CR	25	22	No	Open	12/1315/6	5/15/5	No	7 (28)	0 (0)	0 (0)	0 (0)	64 (2)
	ONU	24	22				0/11/12		8 (33)	0 (0)		2 (8)	58 (2)

Manabe et al. [20]	CR	58	14	Excluded	Open	28/3070/96	4/31/23	No	19 (33)	1 (2)	1 (2)	10 (17)	85 (2)
	ONU	166	28				15/87/64		63 (38)	2 (1)		33 (20)	87 (2)

Taweemonkongsap et al. [13]	CR	31	26	Yes	Open	16/15	18LG/13HG	SD	9 (29)	2 (6)	0 (0)	3 (10)	86 (2)
	ONU	29	28			13/16	10LG/19HG		13 (45)	1 (3)		2 (7)	93 (2)

Chung et al. [14]	HAT	39	48	No	Open	18/21	1/22/16	SD	17 (44)	1 (3)	0 (0)	6 (15)	90 (5)
	ONU	36	60			17/19	1/20/15		13 (36)	3 (8)		4 (11)	86 (5)

Raman et al. [15]	HAT	38	32	Yes	Open (30),	27/9	23LG/15HG	No	11 (29)	1 (3)	0 (0)	2 (6)	81 (5)
	ONU	52	51		TURUO (8)	41/11	33LG/19HG		18 (35)	0 (0)		6 (11)	77 (5)

Wolf et al. [16]	HAT	53	25	Yes	TURUO (23), TV (22), Open (6), LS (2)	38/15	18/9/26	SD	(55)	(8)	0 (0)	(16)	80 (3)

Cannon et al. [17]	HAT	34	14	No	Open	17/18	NL	No	8 (24)	0 (0)	0 (0)	2 (6)	NL

Hsueh et al. [21]	HAR	66	38	No	Open	35/31	1/25/38	No	13 (20)	2 (3)	0 (0)	6 (9)	80 (5)
	ONU	77	54			43/33	3/39/34		19 (25)	2 (3)		12 (16)	75 (5)

Chung et al. [18]	HAR	25	32	No	Open	13/12	1/13/11	No	9 (36)	3 (12)	0 (0)	2 (8)	92 (3)
	ONU	41	62			18/23	1/23/17		13 (32)	3 (7)		5 (12)	92 (3)

Nakashima et al. [19]	HAR	35	23	Yes	Open	22 ≤ T2,13T3	17 ≤ G2,18G3	No	12 (34)	1 (3)	0 (0)	3 (9)	89 (2)
	ONU	38	56			26 ≤ T2,11T3	24 ≤ G2,13G3		21 (55)	1 (3)		2 (5)	91 (2)

**Table 3:** Port-site metastasis following LNU. CR, conventional retroperitoneal; CT, conventional transperitoneal; HAT, hand-assisted transperitoneal; LNU, laparoscopic nephroureterectomy; NL, not listed; TCC, transitional cell carcinoma; TURBT, transurethral resection of bladder tumor.

Author	Surgery	Stage	Retrieval bag	Metastases location	Time to metastasis (mo)	Comments
Ahmed et al. [23]	CT	pT3	No	Widespread	8	

Barrett et al. [24]	CT	pT1	No	Widespread	NL	

Otani et al. [25]	CT	pT3	Yes	Trocar	3	Bag torn; preoperative diagnosis of TCC not known

Ong et al. [26]	CR	pT1	Yes	Trocar	12	Stent perforation in proximal ureter noted at time of LNU

Chueh et al. [27]	HAT	pT2	NL	Trocar	8	Bilateral LNU and TURBT performed in renal transplant patient

Micali et al. [28]	CT	pT3	Yes	NL	3	

Micali et al. [28]	CR	pT3	Yes	NL	15	

Micali et al. [28]	HAT	pT3	Yes	NL	3	

Micali et al. [28]	CR	pT1	No	Trocar	3	Preoperative diagnosis of TCC not known

Micali et al. [28]	CR	pT1	Yes	Trocar	NL	Preoperative diagnosis of TCC not known

Micali et al. [28]	CR	pT2	Yes	Trocar	NL	Preoperative diagnosis of TCC not known

Micali et al. [28]	CR	NL	Yes	Trocar	NL	Preoperative diagnosis of TCC not known

Matsui et al. [29]	CR	pT3	No	Trocar	6	Squamous cell carcinoma

Naderi et al. [30]	CT	pT2	No	Trocar, subcostal wound	3	Required conversion to open surgery secondary to renal vein bleeding

Manabe et al. [20]	CR	NL	NL	Widespread	NL	Urine extravasation secondary to urinary tract obstruction noted preop

Schatteman et al. [9]	CT	pT4	No	Widespread	5	Preoperative diagnosis of TCC not known

Schatteman et al. [9]	CT	pT3	No	Widespread	8	Preoperative diagnosis of TCC not known

Schatteman et al. [9]	CT	pT1	Yes	Widespread	11	
